# Supplementary material for: Reconciling Mining with the Conservation of Cave Biodiversity: A Quantitative Baseline to Help Establish Conservation Priorities
Source: PLoS One. 2016 Dec 20;11(12):e0168348. doi: 10.1371/journal.pone.0168348 (PMC5173368; doi:10.1371/journal.pone.0168348)
Supplement: S1 Dataset — (ZIP) [file pone.0168348.s002.zip › Taxa/Serra Norte/SN_2013/N4WS/Lista N4WS-37.pdf]

CAVIDADE N4WS-0037

Classe	Ordem	Fam/Outros	Gên/Outros	Espécie	S-U	U-S	Zon.
Annelida	Oligochaeta			sp.	3		E
Arachnida	Acari	(Oribatida)		sp.3		X	
Arachnida	Acari	(Parasitengonina)		sp.3		X	
Arachnida	Acari	Acaridae		jovem		X	P
Arachnida	Acari	Anystidae	*Erythracarus*	*nasutus*	X		E
Arachnida	Acari	Argasidae	*Ornithodoros*	sp.1	X		E
Arachnida	Amblypygi	Phrynidae	*Heterophrynus*	*longicornis*	2		E
Arachnida	Araneae	Araneidae		jovem	X		E
Arachnida	Araneae	Ctenidae		jovem	5		E
Arachnida	Araneae	Ochyroceratidae	*Speocera*	sp.1	X		E
Arachnida	Araneae	Pholcidae	*Mesabolivar*	*aurantiacus*		X	E
Arachnida	Araneae	Theraphosidae	aff. *Holothele*	sp.1		1	E
Arachnida	Araneae	Theridiidae	*Theridion*	sp.2		X	E
Arachnida	Araneae	Theridiosomatidae	*Plato*	sp.1	X	X	E/P
Arachnida	Opiliones	Escadabiidae		jovem	X		E
Arachnida	Opiliones	Sclerosomatidae		jovem		X	P
Arachnida	Pseudoscorpiones	Bochicidae		sp.1	X		E
Arachnida	Pseudoscorpiones	Chernetidae	*Spelaeochernes*	sp.1	X	X	E/P
Arachnida	Pseudoscorpiones	Chthoniidae	*Pseudochthonius*	sp.1	X		E
Diplopoda	Polydesmida	Chelodesmidae		sp.1		1	P
Entognatha	Collembola	Isotomidae		sp.1	X		E
Entognatha	Collembola	Paronellidae		sp.1	X		E
Entognatha	Collembola	Paronellidae		sp.4	X	X	E/P
Entognatha	Diplura	Campodeidae		sp.1	X	X	E/P
Insecta	Blattodea			jovem	1		E
Insecta	Coleoptera	Carabidae		jovem	X		E
Insecta	Coleoptera	Scydmaenidae		sp.6		X	P
Insecta	Diptera	Cecidomyiidae	Cecidomyiinae	sp.		X	P
Insecta	Diptera	Ceratopogonidae		jovem	X	X	E/P
Insecta	Diptera	Psychodidae	*Sciopemyia*	*sordellii*	X	X	E/P
Insecta	Diptera	Sciaridae	*Bradysia*	sp.	X		E
Insecta	Diptera	Tipulidae	Tipulinae	sp.	X		E
Insecta	Heteroptera	Reduviidae	Emesinae	sp.1	1		E
Insecta	Heteroptera	Reduviidae	Reduviinae	jovem	5	1	E
Insecta	Homoptera	Cixiidae		jovem		X	E
Insecta	Hymenoptera	Formicidae	*Camponotus*	sp.1	X	X	E/P
Insecta	Hymenoptera	Formicidae	*Hypoponera*	sp.1		X	P
Insecta	Hymenoptera	Formicidae	*Odontomachus*	sp.1	X		E
Insecta	Hymenoptera	Formicidae	*Solenopsis*	sp.1	X		E
Insecta	Hymenoptera	Formicidae	*Solenopsis*	sp.2		X	E
Insecta	Isoptera	Termitidae	*Nasutitermes*	sp.	X	X	E
Insecta	Isoptera	Termitidae	*Spinitermes*	sp.		X	E
Insecta	Isoptera	Termitidae	*Termes*	sp.		X	P
Insecta	Lepidoptera	Noctuoidea		sp.12	5		E
Insecta	Lepidoptera	Tineoidea		sp.3	X		E
Insecta	Neuroptera	Myrmeleontidae		jovem	1	2	E/P
Insecta	Orthoptera	Phalangopsidae	*Paraclodes*	sp.		1	E
Insecta	Orthoptera	Phalangopsidae	*Phalangopsis*	sp.	11	4	E
Insecta	Orthoptera	Phalangopsidae		sp.3		1	E

Insecta	Psocoptera	Psocomorpha		jovem		X	P
Insecta	Psocoptera	Psyllipsocidae		jovem		X	
Malacostraca	Isopoda	Scleropactidae	*Circoniscus*	*buckupi*	X		E
Amphibia	Anura	Strabomantidae	*Pristimantis*	cf. *fenestratus*	2	1	E
